# Risk factors for maternal near-miss in an undeveloped province in south-central China, 2012–2022

**DOI:** 10.1186/s12889-024-18970-4

**Published:** 2024-06-06

**Authors:** Xu Zhou, Junqun Fang, Yinglan Wu, Jie Gao, Xiaoying Chen, Aihua Wang, Chuqiang Shu

**Affiliations:** Hunan Provincial Maternal and Child Health Care Hospital, Changsha, Hunan Province China

**Keywords:** Maternal near-miss, Epidemiology, Risk factor, Advanced maternal age, Cause

## Abstract

**Objective:**

To explore the risk factors for maternal near-miss (MNM) using the WHO near-miss approach.

**Methods:**

Data were obtained from the Maternal Near-Miss Surveillance System in Hunan Province, China, 2012–2022. Multivariate logistic regression analysis (method: Forward, Wald, α = 0.05) and adjusted odds ratios (aORs) were used to identify risk factors for MNM.

**Results:**

Our study included 780,359 women with 731,185 live births, a total of 2461 (0.32%) MNMs, 777,846 (99.68%) non-MNMs, and 52 (0.006%) maternal deaths were identified. The MNM ratio was 3.37‰ (95%CI: 3.23–3.50). Coagulation/hematological dysfunction was the most common cause of MNM (75.66%). Results of multivariate logistic regression analysis showed risk factors for MNM: maternal age > = 30 years old (aOR > 1, *P* < 0.05), unmarried women (aOR = 2.21, 95%CI: 1.71–2.85), number of pregnancies > = 2 (aOR > 1, *P* < 0.05), nulliparity (aOR = 1.51, 95%CI: 1.32–1.72) or parity > = 3 (aOR = 1.95, 95%CI: 1.50–2.55), prenatal examinations < 5 times (aOR = 1.13, 95%CI: 1.01–1.27), and number of cesarean sections was 1 (aOR = 1.83, 95%CI: 1.64–2.04) or > = 2 (aOR = 2.48, 95%CI: 1.99–3.09).

**Conclusion:**

The MNM ratio was relatively low in Hunan Province. Advanced maternal age, unmarried status, a high number of pregnancies, nulliparity or high parity, a low number of prenatal examinations, and cesarean sections were risk factors for MNM. Our study is essential for improving the quality of maternal health care and preventing MNM.

## Introduction

Maternal mortality is an important indicator to evaluate the health status in developing countries [1]. Reducing maternal mortality is one of the priority goals on the international agenda [2]. With the rapid decline in maternal mortality [1, 3, 4], more and more healthcare workers, program managers, and policy-makers responsible for the quality of maternal healthcare are focusing on maternal near-miss (MNM) [5–9]. They expect to describe the epidemiology of MNM, identify risk factors, implement targeted interventions, improve health care, and prevent MNM from developing into maternal deaths.

In 2009, the WHO published the report “Evaluating The Quality of Care for Severe Pregnancy Complications - The WHO Near-miss Approach for Maternal Health” for healthcare workers, program managers, and policy-makers responsible for the quality of maternal healthcare worldwide [10]. It presents a standard approach for monitoring the implementation of critical interventions in maternal health care and proposes a systematic process for assessing the quality of care. In October 2010, China’s National Maternal Near-Miss Surveillance System was established using the WHO near-miss approach, and 18 representative hospitals in Hunan Province are included in the system [11, 12].

Many studies on MNM exist in low- to middle-income countries [7, 13]. However, there are few studies on MNM in China and even fewer multi-factor analyses of influencing factors. The only few studies have samples from the relatively economically developed eastern region or are based on limited data [14–18]. More studies need to be included in China.

Hunan Province is located in south-central China and covers a population of about 65 million. Compared to eastern China, Hunan Province is relatively underdeveloped [19]. In this study, we aim to explore the risk factors for MNM using surveillance data from Hunan Province, 2012–2022.

## Methods

### Data sources

This study used data from the Maternal Near-Miss Surveillance System in Hunan Province, China, 2012–2022. This system uses the WHO near-miss approach [10] in 18 representative registered hospitals in Hunan Province and is run by the Hunan Provincial Health Commission and the China Ministry of Health. Detailed information about the data collection process has been reported elsewhere [11]. In all 18 hospitals, data were collected for all pregnant and post-partum women using an especially designed data collection form. Data were collected for sociodemographic characteristics, obstetric history, place and method of delivery, pregnancy outcome, and complications during pregnancy, delivery, or post-partum. The definition of indicators and collection of information complied with WHO standards [10, 12].

### Informed consents

We confirmed that informed consent was obtained from all subjects and/or their legal guardian(s). Doctors obtain consent from pregnant women before collecting surveillance data, witnessed by their families and the heads of the obstetrics. Since The Hunan Provincial Health Commission collects those data, and the government has emphasized the privacy policy in the “National Maternal Near Miss Surveillance Working Manual”, there is no additional written informed consent.

### Ethics guideline statement

The Medical Ethics Committee of Hunan Provincial Maternal and Child Health Care Hospital approved the study. (NO: 2023-S018). It is a retrospective study of medical records; all data were fully anonymized before we accessed them. Moreover, we de-identified the patient records before analysis. We confirmed that all operations were following relevant guidelines and regulations.

### Data quality control

The Hunan Provincial Health Commission developed the “Maternal Near Miss Surveillance Working Manual” for surveillance. Data were collected and reported by experienced and trained doctors and nurses. To ensure data consistency and accuracy, all collectors must be trained and qualified before starting work. The Hunan Provincial Health Commission asks the technical guidance departments to conduct comprehensive quality control yearly to reduce surveillance data integrity and information error rates.

### Definitions

The following are definitions of MNM indicators according to the WHO near-miss approach [10]. **Maternal death** is the death of a woman while pregnant or within 42 days of termination of pregnancy or its management, but not from accidental or incidental causes. **MNM** refers to a woman who nearly died but survived a complication that occurred during pregnancy, childbirth, or within 42 days of termination of pregnancy. **Non-MNM** refers to a woman who is not a maternal death or MNM case. **A live birth** refers to the birth of an offspring that breathes or shows evidence of life. **MNM ratio** refers to the number of MNM per 1000 live births. Similarly to the severe maternal outcome ratio, this indicator estimates the amount of care and resources needed in an area or facility. **Maternal mortality** refers to the number of maternal deaths per 1000 live births.

### Statistical analysis

MNM ratio, maternal mortality, and its 95% confidence intervals (CI) were calculated by the log-binomial method [20]. Univariate analysis and unadjusted odds ratios (uORs) were used to examine the association of each demographic characteristic with MNM. Multivariate logistic regression analysis (method: Forward, Wald, α = 0.05) and adjusted odds ratios (aORs) were used to identify risk factors for MNM. We used the presence or absence of MNM as the dependent variable, and the variables assessed significantly in univariate analysis were entered as independent variables in multivariate logistic regression analysis.

Statistical analyses were performed using SPSS 18.0 (IBM Corp., NY, USA).

## Results

### MNM ratios in hunan province, China, 2012–2022

**Fig. 1:**
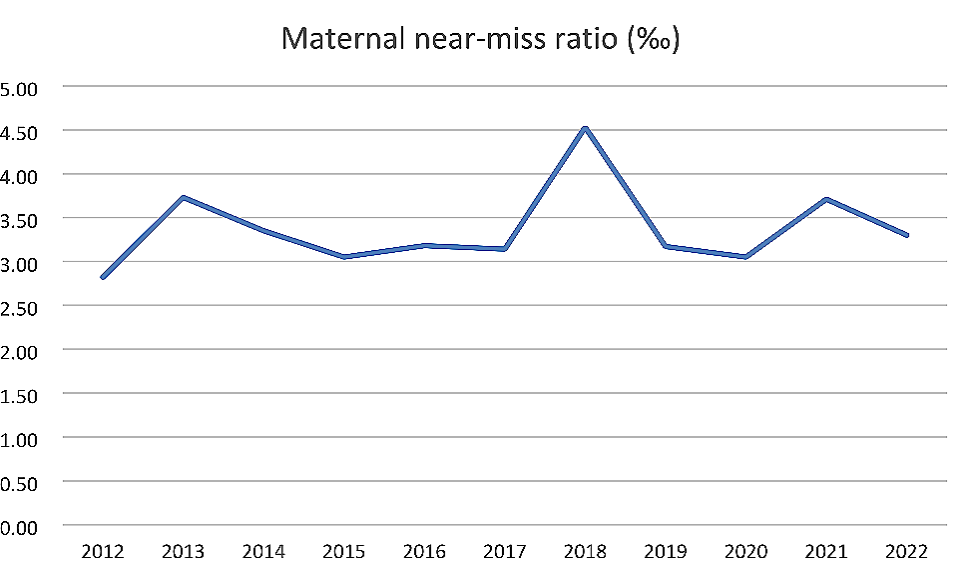
Maternal near-miss ratios in Hunan Province, China, 2012–2022

Our study included 780,359 women with 731,185 live births, a total of 2461 (0.32%) MNMs, 777,846 (99.68%) non-MNMs, and 52 (0.006%) maternal deaths were identified. The MNM ratio was 3.37‰ (95%CI: 3.23–3.50), and the maternal mortality was 0.07‰ (95%CI: 0.05–0.09). Figure 1 and Table 1 show the details of MNM ratios by year. (Figs. 1 and Table 1)

**Table 1:** Basic information on surveillance data in Hunan Province, China, 2012–2022

Year	Total women (*n*)	Live births (*n*)	Non-MNMs (*n*)	MNMs		Maternal deaths	
				*n*	MNM ratio (‰, 95%CI)	*n*	Maternal mortality (‰, 95%CI)
2012	62,608	62,150	62,424	175	2.82(2.40–3.23)	9	0.14(0.05–0.24)
2013	67,886	66,035	67,631	246	3.73(3.26–4.19)	9	0.14(0.05–0.23)
2014	75,817	73,104	75,562	245	3.35(2.93–3.77)	10	0.14(0.05–0.22)
2015	79,513	77,123	79,274	235	3.05(2.66–3.44)	4	0.05(0.00-0.10)
2016	71,310	68,628	71,088	218	3.18(2.75–3.60)	4	0.06(0.00-0.12)
2017	78,919	75,573	78,674	237	3.14(2.74–3.54)	8	0.11(0.03–0.18)
2018	76,647	71,589	76,321	324	4.53(4.03–5.02)	2	0.03(-0.01-0.07)
2019	75,033	68,054	74,814	216	3.17(2.75–3.60)	3	0.04(-0.01-0.09)
2020	68,756	61,926	68,566	189	3.05(2.62–3.49)	1	0.02(-0.02-0.05)
2021	64,101	55,820	63,893	207	3.71(3.20–4.21)	1	0.02(-0.02-0.05)
2022	59,769	51,183	59,599	169	3.30(2.80–3.80)	1	0.02(-0.02-0.06)
Total	780,359	731,185	777,846	2461	3.37(3.23–3.50)	52	0.07(0.05–0.09)

*Abbreviations* MNM: Maternal near-miss; CI: Confidence intervals

### Causes of MNM

Coagulation/hematological dysfunction was the most common cause of MNM (75.66%), followed by cardiovascular dysfunction (23.41%). Hepatic dysfunction was the least common cause of MNM (1.46%). Of the 2461 MNMs, 11.09% were complicated with uterine dysfunction, 10.20% were complicated with neurological dysfunction, 5.44% were complicated with respiratory dysfunction, and 2.03% were complicated with renal dysfunction. Table 2 shows the details of the causes of MNM. (Table 2)

**Table 2:** Causes of MNM

Causes	*n*	Proportion (%)
**Coagulation/hematological dysfunction**	**1862**	**75.66**
Transfusion of > = 5 units of blood/red cells	1613	65.54
Acute thrombocytopenia (< 50 000 platelets)	280	11.38
Clotting failure	226	9.18
**Cardiovascular dysfunction**	**576**	**23.41**
Shock	552	22.43
Use of continuous vasoactive drugs	117	4.75
Lactic acidosis (pH < 7.1, lactate > 5 mmol/L)	46	1.87
Cardio-pulmonary resuscitation	23	0.93
Cardiac arrest	15	0.61
**Uterine dysfunction**	**273**	**11.09**
Hysterectomy due to infection or hemorrhage	273	11.09
**Neurological dysfunction**	**251**	**10.20**
Metabolic coma (loss of consciousness and the presence of glucose and ketoacids in urine)	230	9.35
Coma/loss of consciousness lasting 12 h or more	17	0.69
Status epilepticus/Uncontrollable fits/total paralysis	14	0.57
Stroke	6	0.24
**Respiratory dysfunction**	**134**	**5.44**
Severe hypoxemia (Oxygen saturation < 90% for ≥ 60 min, or PaO2/FiO2 < 200 mmHg)	68	2.76
Intubation and ventilation not related to anesthesia	60	2.44
Acute cyanosis	35	1.42
Respiratory rate > 40/min	33	1.34
Respiratory rate < 6/min	6	0.24
**Renal dysfunction**	**50**	**2.03**
Severe acute azotemia (Creatinine > = 300 mmol/L or > = 3.5 mg/dL)	29	1.18
Oliguria non responsive to fluids or diuretics	24	0.98
Dialysis for acute renal failure	20	0.81
**Hepatic dysfunction**	**36**	**1.46**
Hyperbilirubinemia (Bilirubin > 100 mmol/L or > 6.0 mg/dL)	30	1.22
Jaundice in the presence of pre-eclampsia	12	0.49

*Abbreviations* MNM: Maternal near-miss; N: Number of maternal near-miss cases; CI: Confidence intervals

*Note* The number of total MNMs is 2461

### Results of univariate analysis and multivariate logistic regression analysis for risk factors for MNM

In the univariate analysis, all variables were associated with MNM. Therefore, all variables in Table 3 were entered as independent variables in the multivariate logistic regression analysis. As a result, multivariate logistic regression analyses showed that all variables were also associated with MNM.

**Table 3:** Results of univariate analysis and multivariate logistic regression analysis for risk factors for MNM

Variables	Total women (not including maternal deaths) (*n*)	MNMs (*n*)	Proportion (%)	uOR(95%CI)	aOR(95%CI)
Maternal age (years old)					
< 20	9688	28	0.29	1.22(0.84–1.78)	0.98(0.65–1.47)
20–24	112,201	212	0.19	0.80(0.69–0.93)	0.84(0.72–0.99)
25–29	313,157	741	0.24	Reference	Reference
30–34	234,848	816	0.35	1.47(1.33–1.62)	1.25(1.12–1.38)
>=35	110,413	664	0.60	2.55(2.30–2.83)	1.78(1.58-2.00)
Marital status					
Married	768,591	2392	0.31	Reference	Reference
Unmarried (including single, divorced, widowed, cohabiting, and other)	11,716	69	0.59	1.90(1.49–2.41)	2.21(1.71–2.85)
Number of pregnancies (including the present pregnancy)					
1 (First)	269,109	552	0.21	Reference	Reference
2	228,806	557	0.24	1.19(1.06–1.34)	1.20(1.04–1.37)
3	144,184	555	0.38	1.88(1.67–2.12)	1.71(1.47–1.98)
4	79,393	378	0.48	2.33(2.04–2.65)	1.89(1.59–2.23)
>=5	58,815	419	0.71	3.49(3.07–3.96)	2.41(2.03–2.87)
Parity (Not including the present pregnancy)					
0 (Nulliparity)	400,231	962	0.24	0.68(0.63–0.75)	1.51(1.32–1.72)
1	328,503	1152	0.35	Reference	Reference
2	45,318	279	0.62	1.76(1.54–2.01)	1.16(0.98–1.37)
>=3	6255	68	1.09	3.12(2.44–3.99)	1.95(1.50–2.55)
Prenatal examinations (times)					
< 5	103,934	416	0.40	1.20(1.08–1.34)	1.13(1.01–1.27)
5–7	222,693	596	0.27	0.80(0.73–0.89)	0.83(0.75–0.92)
8–10	401,886	1337	0.33	Reference	Reference
>=11	51,794	112	0.22	0.65(0.54–0.79)	0.65(0.54–0.79)
Number of cesarean sections (not including the present pregnancy)					
0 (No cesarean section)	634,900	1617	0.25	Reference	Reference
1	133,213	705	0.53	2.08(1.91–2.28)	1.83(1.64–2.04)
>=2	12,194	139	1.14	4.52(3.79–5.38)	2.48(1.99–3.09)
Total	780,307	2461	0.32	-	-

*Abbreviations* MNM: Maternal near-miss; uOR = unadjusted odds ratio; aOR = adjusted odds ratio; CI = confidence intervals

MNMs were more common in unmarried women than married women (aOR = 2.21, 95%CI: 1.71–2.85). Compared to maternal age 25–29 years old, MNMs were more common in maternal age 30–34 years old (aOR = 1.25, 95%CI: 1.12–1.38) or > = 35 years old (aOR = 1.78, 95%CI: 1.58-2.00). Compared to the first pregnancy, MNMs were more common in number of pregnancies was 2 (aOR = 1.20, 95%CI: 1.04–1.37), or 3 (aOR = 1.71, 95%CI: 1.47–1.98), or 4 (aOR = 1.89, 95%CI: 1.59–2.23), or > = 5 (aOR = 2.41, 95%CI: 2.03–2.87), and the aOR values showed an upward trend. Compared to parity was 1, MNMs were more common in parity was 0 (aOR = 1.51, 95%CI: 1.32–1.72), or 2 (aOR = 1.16, 95%CI: 0.98–1.37), or > = 3 (aOR = 1.95, 95%CI: 1.50–2.55). Compared to prenatal examinations were 8–10 times, MNMs were more common in prenatal examinations < 5 times (aOR = 1.13, 95%CI: 1.01–1.27) or less common in 5–7 times (aOR = 0.83, 95%CI: 0.75–0.92) or > = 11 times (aOR = 0.65, 95%CI: 0.54–0.79). Compared to no cesarean section, MNMs were more common in the number of cesarean sections was 1 (aOR = 1.83, 95%CI: 1.64–2.04) or > = 2 (aOR = 2.48, 95%CI: 1.99–3.09).

There were significant differences in the results of univariate analysis and multivariate logistic regression analysis for some factors. For example, nulliparity is a protective factor for MNM in the univariate analysis (uOR = 0.68, 95%CI: 0.63–0.75), while a risk factor in the multivariate logistic regression analysis (aOR = 1.51, 95%CI: 1.32–1.72). In addition, there were significant differences between uOR and aOR values for some factors. For example, the parity > = 3 (uOR = 3.12, aOR = 1.95), and the number of cesarean sections > = 2 (uOR = 4.52, aOR = 2.38). Table 3 shows the details of univariate analysis and multivariate logistic regression analysis (Table 3).

## Discussion

Our study is the first systematic study of risk factors for MNM at the provincial level in a relatively undeveloped province in China [7, 14]. Advanced maternal age, unmarried status, a high number of pregnancies, nulliparity or parity > = 3, prenatal examinations < 5 times, and cesarean section were risk factors for MNM.

In this study, the MNM ratio was 3.37‰, lower than most middle-income countries (9.6‰, interquartile range: 7.0–23.3) [7], and lower than several other regions of China. For example, the MNM ratio was 5.9‰ in Zhejiang Province (2012–2017) [14], 3.81‰ in a hospital in Suzhou, Jiangsu Province (2008–2012) [21], 12.4‰ in a hospital in Hefei, Anhui Province (2012–2015) [17]. In addition, maternal mortality (0.07‰) was lower than in most middle-income countries (163 per 100 000 live births, interquartile range: 52–367) [7]. However, the maternal mortality or MNM ratio was higher than in some developed regions of China or some developed countries. For example, The maternal mortality in Zhejiang Province was 5.6 per 100,000 live births [14]. The MNM ratio was 1.8‰ in Ireland [22] and 2.0‰ in Italy [23]. It indicates that there is still room for improvement in the quality of maternal health care. In addition, from 2012 to 2022, the MNM ratio changes significantly. For example, the MNM ratio was 4.53‰ in 2018 and 2.82‰ in 2012.

The above findings may be related to several factors. First, the variation across countries may be mainly associated with economic and medical conditions. Economic and medical conditions may influence the quality of care, and better economic and medical conditions are associated with lower MNM ratios [7]. Second, the variation across years and regions in China may be associated with several other factors, such as the “two-child policy” in China in 2015 [24], the Healthy China 2030 national strategy in 2016 [25], the high prevalence of hypertensive disorders in pregnancy [26], gestational diabetes [27] and obesity [28]. In addition, some demographic characteristics of pregnant women may also significantly impact the causes of MNM, as shown by the multivariate logistic regression analyses.

Overall, the MNM ratio in this study was relatively low, and maternal mortality decreased significantly from 2012 to 2022. The Hunan Provincial Statistical Yearbook shows a steady development of economic and medical conditions in Hunan Province from 2012 to 2022 [19]. It indicates that the quality of maternal health care in Hunan Province is relatively high.

In this study, coagulation/hematological dysfunction was the most common cause of MNM, followed by cardiovascular dysfunction. It is consistent with several previous studies reported in Egypt [29], Pakistani [30], Nigeria [31], Papua New Guinea [32], Kenya [33], and Zhejiang Province (Eastern China) [14]. However, some previous studies were inconsistent with this study. For example, in India [34], Ghana [35], Brazil [36], Iraq [37], and South Africa [38], the most common cause of MNM was hypertensive disorders. In addition, some of the specific causes of MNM in this study differed from previous studies. For example, in our study, hemorrhage was the most common specific cause of MNM (65.54%), which was significantly higher than the 26% reported by the WHO multi-country Survey [39]. As specific causes of MNM, renal dysfunction (2.03% vs. 1.3%) and neurological dysfunction (10.20% vs. 7.7%) were more common in this study than in Zhejiang Province. Respiratory dysfunction (5.44% vs. 8.6%) and uterine dysfunction (11.09% vs. 14.6%) were less common in this study than in Zhejiang Province [14]. Similar to the previous discussion, it may be associated with several factors, such as economic and medical conditions.

We have identified several risk factors for MNM. First, advanced maternal age was a risk factor for MNM, consistent with many previous studies [14, 40–42]. It is associated with the fact that advanced maternal age is at a higher risk of adverse obstetrical and perinatal outcomes [43]. We believe that nulliparity or parity > = 3 and cesarean section were risk factors for MNM, which may also be associated with adverse obstetrical and perinatal outcomes. For example, previous studies have shown that cesarean section was associated with advanced maternal age [44], high parity was associated with some medical complications and placental pathologies [45, 46], and nulliparity was associated with adverse pregnancy outcomes and MNM [47–49].

Second, unmarried status was a risk factor for MNM. It may be mainly associated with worse economic and medical conditions and poor mental health among unmarried women, which have a significant impact on the causes of MNM (similar to the above discussion). In this study, about half of the unmarried women were single, and the others were widowed or divorced. Those unmarried women may have lower quality of care, may easily suffer from mental disorders, and may face many life-threatening situations during pregnancy [50, 51]. We believe the increased risk of MNM for prenatal examinations < 5 times may also be associated with worse economic and medical conditions. In Hunan Province, China, pregnant women receive 8–10 times of prenatal examinations on a regular schedule as required. Regular prenatal examinations allow most women with pregnancy complications to be detected and treated in time, which may reduce the MNM ratio. On the contrary, inadequate and irregular prenatal examinations may increase the MNM ratio [52].

Third, a high number of pregnancies was a risk factor for MNM. Previous studies have shown that a high number of pregnancies may be mainly associated with spontaneous miscarriages, many of which were recurrent miscarriages, and may be associated with disorders such as chromosomal abnormalities [53–55]. Miscarriages can induce pronounced emotional responses, such as anxiety, depression, denial, anger, marital disruption, and a sense of loss and inadequacy [53, 56]. These conditions may be associated with adverse obstetrical and perinatal outcomes.

In addition, the results of some previous studies were inconsistent with this study. For example, Yang et al. found that the number of pregnancies was not associated with MNM [17]. Nik et al. found that the sole risk factor for MNM was a history of cesarean Sect. [57]. It may be associated with confounding factors. Some demographic characteristics were also not included in this study.

Some things could be improved in this study. First, due to data limitations, some demographic characteristics, such as economic conditions, were not included in this study. Second, although some meaningful results were found, the associations between risk factors and MNM showed only correlation and may not be causal. The mechanisms should be further investigated. Third, there may be the risk of under-reporting MNMs in the surveillance system, especially at some county-level surveillance sites.

## Conclusion

The MNM ratio was relatively low in Hunan Province. Advanced maternal age, unmarried status, a high number of pregnancies, nulliparity or high parity, a low number of prenatal examinations, and cesarean sections were risk factors for MNM. Our study is essential for improving the quality of maternal health care and preventing MNM.

## Data Availability

All data generated or analyzed during this study are included in this published article.

## Abbreviations

MNM: Maternal Near-Miss
CI: Confidence Intervals
OR: Odds Ratios
